# High-throughput analysis of *Yersinia pseudotuberculosis* gene essentiality in optimised in vitro conditions, and implications for the speciation of *Yersinia pestis*

**DOI:** 10.1186/s12866-018-1189-5

**Published:** 2018-05-31

**Authors:** Samuel J. Willcocks, Richard A. Stabler, Helen S. Atkins, Petra F. Oyston, Brendan W. Wren

**Affiliations:** 1 Department of Pathogen Molecular Biology, London School of Hygiene and Tropical Medicine, Keppel Street, London, WC1E 7HT UK; 2 Microbiology, CBR Division, DSTL Porton Down, Salisbury, SP4 0JQ UK

**Keywords:** *Yersinia*, TraDIS, TnSeq, Essential genes

## Abstract

**Background:**

*Yersinia pseudotuberculosis* is a zoonotic pathogen, causing mild gastrointestinal infection in humans. From this comparatively benign pathogenic species emerged the highly virulent plague bacillus, *Yersinia pestis*, which has experienced significant genetic divergence in a relatively short time span. Much of our knowledge of *Yersinia spp.* evolution stems from genomic comparison and gene expression studies. Here we apply transposon-directed insertion site sequencing (TraDIS) to describe the essential gene set of *Y. pseudotuberculosis* IP32953 in optimised in vitro growth conditions, and contrast these with the published essential genes of *Y. pestis*.

**Results:**

The essential genes of an organism are the core genetic elements required for basic survival processes in a given growth condition, and are therefore attractive targets for antimicrobials. One such gene we identified is *yptb3665*, which encodes a peptide deformylase, and here we report for the first time, the sensitivity of *Y. pseudotuberculosis* to actinonin, a deformylase inhibitor. Comparison of the essential genes of *Y. pseudotuberculosis* with those of *Y. pestis* revealed the genes whose importance are shared by both species, as well as genes that were differentially required for growth. In particular, we find that the two species uniquely rely upon different iron acquisition and respiratory metabolic pathways under similar in vitro conditions.

**Conclusions:**

The discovery of uniquely essential genes between the closely related *Yersinia spp.* represent some of the fundamental, species-defining points of divergence that arose during the evolution of *Y. pestis* from its ancestor. Furthermore, the shared essential genes represent ideal candidates for the development of novel antimicrobials against both species.

**Electronic supplementary material:**

The online version of this article (10.1186/s12866-018-1189-5) contains supplementary material, which is available to authorized users.

## Background

*Yersinia pseudotuberculosis* can colonise a wide range of animal and bird hosts, from which human infection can arise causing gastrointestinal illness. It is an important disease of young cattle in particular, and can result in chronic morbidity, or acute septicaemia, haemorrhagic diarrhoea and death [1]. The difficulty in diagnosing yersiniosis in both cattle and humans means that cases are likely to be considerably underreported [2, 3].

The study of *Y. pseudotuberculosis* has added significance because of its genetic similarity to the plague bacillus, *Yersinia pestis*, a recently emerged and highly virulent species [4]. The common ancestor of *Y. pestis* that is capable of causing bubonic plague is thought to have emerged in the vicinity of China some 5700 years ago [5, 6]. That strain is itself thought to have emerged from a less virulent ancestor that diverged from *Y. pseudotuberculosis* about 54,000 years ago.

The vast majority of genes present in *Y. pseudotuberculosis* share over 97% identity with the respective orthologues in *Y. pestis* [7]. While genetic conservation between the two species can be used to explain shared characteristics, genetic differences can help inform us of the evolution of virulence in *Y. pestis*. The known genetic differences between *Y. pestis* and *Y. pseudotuberculosis* to date have been described as the “foothold moments” that mark the emergence of the highly virulent species from its ancestor [8]. Such differences may include, for example, the acquisition of the plasmids pMT1 and pPCP1 by *Y. pestis* [9], as well gene inactivation by negative transcriptional regulation [10] and genome size reduction [11]. Whole genome sequencing has revealed that *Y. pestis* has approximately 100,000 fewer nucleotides than its immediate ancestor [11]. The accumulation of pseudogenes further contributes to the ongoing divergence. For example, *Y. pseudotuberculosis* IP32953 has a reported 62 pseudogenes compared to over 200 pseudogenes in strains of *Y. pestis* [7].

Gene loss may not necessarily result in a loss of function, but can sometimes facilitate the gain of a new capability. For example, *Y. pestis* has a mutation compared with *Y. pseudotuberculosis* that prevents the formation of O-antigen in its lipopolysaccharide (LPS) [12]. However, the lack of O-antigen facilitates the activity of plasminogen activator, a plasmid-borne virulence factor found in strains of *Y. pestis* causing bubonic plague [13].

*Y. pestis* and *Y. pseudotuberculosis* occupy different niches that afford unique resources and challenges, and hence the absolute requirement of certain genes changes. For example, while *Y. pseudotuberculosis* is well adapted to survival in the soil, *Y. pestis* is less fit in this environment [14]. While both are capable of infecting mammals, the infection cycle of *Y. pestis* does not require survival in the mammalian gut, whereas it does require reaching a high cell density to cause bacteraemia [15], and can also cause pneumonic infection [16]. Finally, only *Y. pestis* is fully adapted for survival in and transmission by the flea vector [14, 17, 18]. The genetic differences between *Y. pestis* and *Y. pseudotuberculosis* that contributed to vector-borne transmission and enhanced virulence in humans are well studied, and it can be concluded that following successful adaptation of *Y. pestis* to its new niche, detrimental or superfluous genes were gradually lost [19]. Using Transposon-Directed Insertion-site Sequencing (TraDIS), also known as Transposon Sequencing (TnSeq), we are able to identify such genes in unprecedented detail.

TraDIS, or TnSeq, was first demonstrated in *Salmonella* Typhi [20], and has subsequently been applied to several pathogenic species including *Vibrio cholerae* [21], *Escherichia coli* [22] and *Burkholderia pseudomallei* [23]. Libraries are constructed from individual randomly-integrated transposon mutants that are then pooled, ranging in complexity from the small, (< 3000 [24]), to the very large, (in excess of 1,000,000 [23]) unique transposon mutants, saturating the genome several times over. Negative selection of transposon insertion mutants reveals their essentiality in the chosen in vitro or in vivo condition. In this study, we utilised TraDIS to establish the functional essentiality of every gene in the *Y. pseudotuberculosis* IP32953 genome under optimised in vitro growth conditions. This is in contrast to previous *Y. pseudotuberculosis* TraDIS studies that have focused on the discussion of in vivo virulence-associated genes [25].

Essential genes, particularly those that are conserved among species but with no orthologue in humans, represent potential targets for novel or re-purposed antibiotics. In the present work, we demonstrate the efficacy of actinonin, a deformylase inhibitor, against *Y. pseudotuberculosis* based on the discovery that peptide deformylase, *yptb3665*, is required for growth in vitro. Furthermore, we exploit the recently published *Y. pestis* essential gene data [26] and compare this to our *Y. pseudotuberculosis* TraDIS. This allowed us to contrast the differing importance of genes required for growth of either *Yersinia spp.* in optimised nutritional conditions. A strength of this approach is that it is not limited to only identifying the genes that are present or absent between the species, which has informed most of our knowledge of *Yersinia spp.* pathogen evolution to date. Also, unlike transcriptional profiling data, in which not all transcribed genes are essential, TraDIS identifies the absolute necessity of all the genes required for survival. Thus, we are able to report not only the conserved genes between *Y. pseudotuberculosis* and *Y. pestis* that are essential for growth, but also where gene essentiality has been lost, and where new genes have taken on greater significance in *Y. pestis*. These represent a record of the key sites of divergence that resulted from the emergence of *Y. pestis* from *Y. pseudotuberculosis*.

## Results

The Ez-Tn*5*-Kan transposome complex was used to generate a library of approximately 40,000 mutants in *Y. pseudotuberculosis* IP32953. DNA sequencing of our ‘wild-type’ laboratory isolate of IP32953 revealed three non-synonymous amino-acid mutations compared with the reference sequence, these were located within: *celB*, a carbohydrate transporter; *yptb3499*, an uncharacterised gene and *yptb0420*, an uncharacterised gene.

**Fig. 1:**
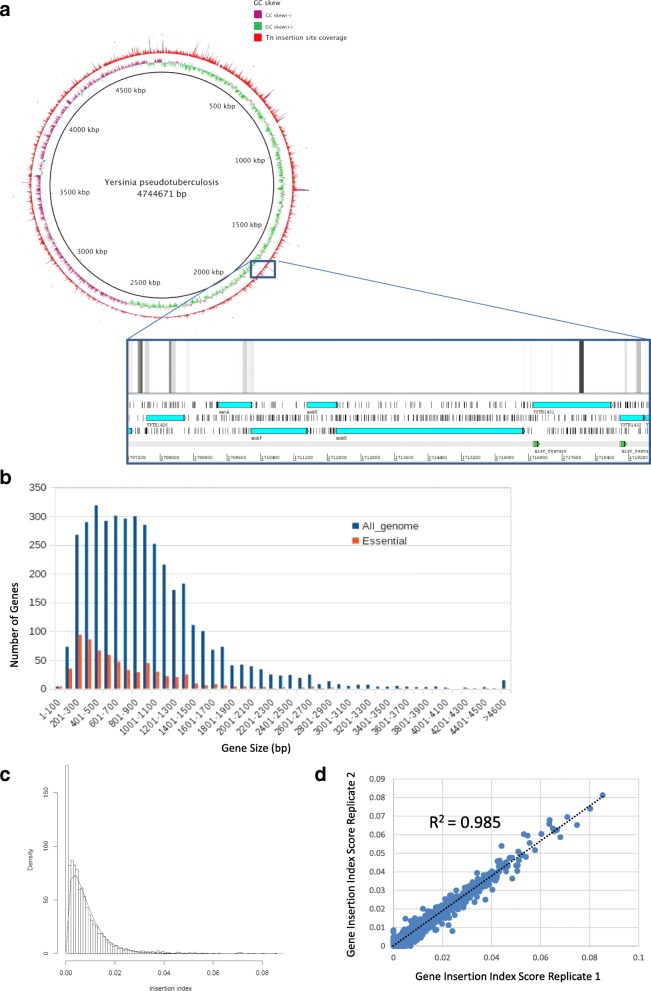
**a** Infographic showing the complete coverage of ~ 40,000 transposon insertions of Tn5 into the *Y.pseudotuberculosis* chromosome from the pooled TraDIS library. Transposon insertions (RED) occur evenly throughout the genome, with no obvious bias relating to high (GREEN) or low (PURPLE) GC content. Depth of reads are indicated by the height of the bars; gaps in coverage represent the location of essential genes. Inset: presence of essential genes at the *muk* gene cluster as visualised using Artemis software; heatmap of transposon insertions are mapped against the reference genome. Ring image generated using BRIG Blast Ring Image Generator. **b** Histogram showing gene-size distribution of the *Y. pseudotuberculosis* genome alongside gene-size distribution of identified essential genes. **c** Gamma-curve distribution revealing the insertion-index threshold value at which genes contain no, or very few insertions are considered to be essential. **d** Correlation coefficient between two technical replicates of TraDIS. Gene insertion index scores for every gene were compared between replicates and plotted; the coefficient score was determined using CORREL function in Microsoft Excel

Transposon coverage was mostly evenly distributed throughout the chromosome, although there was a lower density of transposon insertions between 2 and 3000 Kbps. We did not observe any apparent bias toward GC content or towards the origin of replication (Fig. 1a). In general, the distribution of gene sizes identified as essential reflected the distribution of gene sizes for the genome as a whole (Fig. 1b). However, below 200 bp a higher proportion of genes were identified as essential, suggesting their small size reduced the likelihood of transposon insertion regardless of gene function. This aspect was taken into account by generating an insertion index during TraDIS data analysis as described by others [20, 27–29], which normalised insertion frequency against gene length for every gene; a low gene insertion index score is indicative of increased likelihood of essentiality. A gamma distribution curve was generated to visualise the data and calculate the threshold value at which a given gene was considered to be essential (Fig. 1c). As a quality control step, we generated a correlation coefficient between two technical replicates of the TraDIS, showing the reproducibility of the gene insertion index between replicates across the whole genome (Fig. 1d).

We generated 566,387 high-quality reads with confirmed transposon barcoding, of which 297,216 reads mapped to the reference sequence. Un-mapped reads were accounted for by *phi*X DNA used to facilitate sequencing; genomic DNA clusters that did not contain transposon; and reads that contained at least one mismatch with the transposon sequence. We used a custom-designed single-primer adenylation method [30] to reduce non-unique PCR amplification bias during sequencing preparation, which we conservatively limited to 10 cycles.

For assessing essentiality, we pooled the collective data from three independent sequencing reactions. The *Y. pseudotuberculosis* genome contains 4324 predicted coding sequences (CDSs), excluding non-coding RNA genes. We identified 24,480 unique genome insertion events, representing, on average, seven transposon insertions per gene, of which 3796 were disrupted by transposon insertion. An example of essential genes showing lack of transposon insertion is provided in the enlarged inset of Fig. 1a: the *mukE/F/B* operon, involved in chromosome condensation, segregation and cell-cycle progression.

From the resulting 534 CDSs that we identified as essential in our experimental conditions, we excluded 46 genes belonging to known phage-related genes, leaving 488 in vitro growth essential genes (Additional file 1: Table S1).

**Fig. 2:**
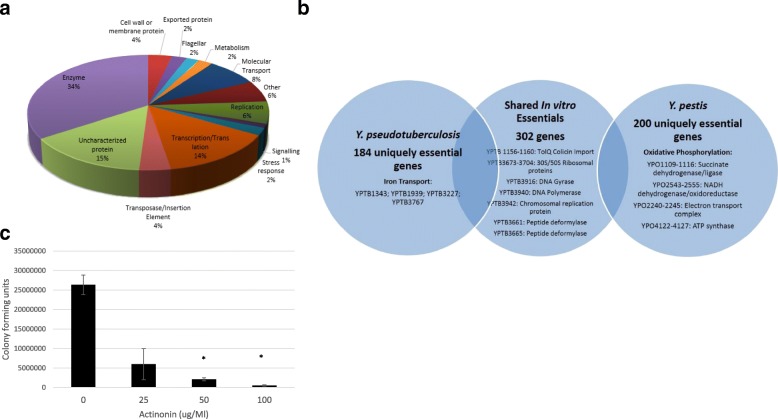
**a** Functional Distribution of Essential Genes. The essential genes of *Y. pseudotuberculosis* were qualitatively assessed and grouped for their predicted function, excluding phage-related genes. **b** Venn Diagram depicting relationship between genes that were essential for in vitro growth in both *Y. pseudotuberculosis* and *Y. pestis*, and those that were found to be uniquely essential to either species. Examples of genes are provided in each condition. **c** Essential Genes Can Be Used to Predict Antibiotic Sensitivity. *Y. pseudotuberculosis* IP32953 in BAB growth media was incubated at 28 °C with a titrated dose of actinonin, prior to enumeration of colony forming units. Student’s T-test conducted versus the untreated condition (* = *p* < 0.01)

The genes we identified from in vitro growth of *Y. pseudotuberculosis* IP32953 in liquid BAB media at 28 °C are dominated by genes encoding proteins with enzymatic function, of which kinases, transferases and synthases are highly represented (summarised in Fig. 2a). The next largest group contains genes encoding proteins involved in transcription, translation and replication. Metabolic function and transporters are also a significant subset of the essential genes. Notably, several large clusters are identified such as the *hem* and *rpl* genes and those with related function. *Rpl* genes encode the 50S ribosomal proteins, and *hem* is involved in the haeme porphyrin biosynthesis pathway.

By comparing our findings with the published *Y. pestis* in vitro essential genes [26], cultured using identical methods, we were able to identify shared and contrasting essential genes between the species (Additional file 2: Table S2). A Venn diagram showing selected shared and unique essential genes between *Y. pseudotuberculosis* and *Y. pestis* highlights some prominent trends, such differences relating to iron acquisition and oxidative phosphorylation (Fig. 2b).

We were interested to test the hypothesis that essential genes may represent targets for specific compounds that may be bactericidal. We identified *yptb3665*, which encodes a peptide deformylase-type enzyme, and subsequently identified that *Y. pseudotuberculosis* was sensitive to the deformylase inhibitor, actinonin (Fig. 2c).

## Discussion

### The essential gene set of *Y. pseudotuberculosis* IP32953

Previous *Y. pseudotuberculosis* transposon library screens have focused on identifying virulence genes [25, 31–33], and as such have used smaller transposon libraries that are appropriate for in vivo studies. In this study, we have screened a more complex library for essential genes required for growth in complex laboratory media.

The 485 in vitro growth essential genes we identified is comparable with other pathogens that have been assessed using similar methods; *B. pseudomallei* is reported to have 505 essential genes [23]; *Haemophilus influenza* has 532 [34]; 636 in *Pseudomonas aeruginosa* [35]; 353 in *Salmonella* Typhimurium [36] and *Clostridium difficile* possesses 404 essential genes [37]. A large proportion of essential genes we identified have as-yet uncharacterised function, representing a limitation in our understanding of the pathogen.

Some artefact genes were identified such as those encoding flagellar proteins, mutation of which may lower competitive fitness in liquid broth conditions without being true essential genes. Similarly, some genes have been reported to tolerate insertions at the three-prime region without complete loss of function [26]. While the risk of naming false essential genes can be reduced through adequate library complexity, biological replicates and the use of insertion-index scores, we acknowledge a margin of error inherent in any TraDIS/Tnseq study. This stems partly from the difficulty in imposing binary bioinformatics-based thresholds of ‘essentiality’ on a biological system that instead operates in non-discrete degrees of fitness.

### TraDIS-informed identification of potential drug targets

The identification of essential genes can contribute to the identification of novel antibacterial drug targets. Two types of essential genes have been proposed as novel targets for antimicrobial development: in vivo essential, or ‘virulence’ genes, and truly essential genes that are required for viability [38]. Whilst virulence genes make attractive targets for specific pathogens, any inhibitors are narrow in their selectivity and thus there is a requirement for companion diagnostics to guide clinical use. In contrast, truly essential genes are often involved in conserved processes, and as such may represent opportunities for broader spectrum therapeutics to be developed.

To minimise potential host-toxicity, bacterial essential genes that have no orthologue in mammals are attractive targets. In our TraDIS screen, we identified *yptb3665*, which encodes a peptide deformylase-type enzyme that appear to be essential for bacterial growth but are not found in eukaryotic cells [39]. They are the subject of a new class of inhibitor that shows promising activity and safety in vivo [40–42]. We hypothesised that since *yptb3665* was found to be essential in both *Y. pseudotuberculosis* IP32953 and *Y. pestis* CO92 (*ypo0240*), then exposure to specific inhibitors should reduce viability. We were able to demonstrate that one member of this class antibiotic, actinonin, was indeed effective in inhibiting *Y. pseudotuberculosis*. Although *yptb3665* itself is not widely conserved outside the genus, it is conserved among *Y. pseudotuberculosis* strains. Actinonin and related compounds have shown activity against various other deformylases across a wide range of bacteria, including *E. coli*, *Staphylococcus aureus*, *Haemophilus influenzae* and *Streptococcus pneumonia* [43, 44], but to our knowledge this is the first study demonstrating that a peptide deformylase is essential in a *Yersinia* species, and that *Y. pseudotuberculosis* it is sensitive to a peptide deformylase inhibitor. Further investigation would be required to prove that yptb3665 is the specific target of actinonin.

### Identification of phage and associated genes by TraDIS

An unexpected finding in our TraDIS ‘essential’ dataset are the numerous genes encoding bacteriophage proteins. We do not consider it likely that genes encoding structural phage proteins such as the capsid or tail are related to bacterial fitness, although interestingly, a TnSeq study of bacteriophage similarly found that some genes encoding structural proteins resisted transposon insertion [45]. Our findings may partly be an artefact due to several of the phage genes having very short sequences, making them less likely to receive a transposon insertion. However, given the level of saturation we achieved in the library, it is still surprising that we identify so many phage genes. A number of the phage genes are responsible for the maintenance of lysogeny, interruption of which may trigger entry into the lytic cycle [46], and indeed these have been identified in previous TraDIS screens by others [35, 36, 47]. Interruption of any gene required for phage persistence or replication would also result in its absence from the overall population. Since many of these phage genes are part of a single open reading frame, transposon insertion into a non-essential gene may influence the transcription of these downstream regulatory genes.

Chromosomal DNA-protection mechanisms may also affect transposon insertion efficiency. For example, among the essential genes we identified a putative chromosomal phage toxin-antitoxin (TA) system comprised of death on curing protein (*yptb1707*) and prevent host-death protein, predicted to be *yptb1706.* Chromosomally integrated TA systems have been suggested as contributing to the genetic stability and maintenance of neighbouring genes at the locus [48–50]. We also identified predicted host restriction modification (RM) systems (*yptb3879* and *yptb3881*) close to the phage regions. RM systems have been described as selfish genetic elements that may resist the integration of exogenous DNA by killing the host cell [51]. In addition, non-methylated transposon DNA may be susceptible to degradation by the RM at these loci [52]. Phage-encoded RM systems (potentially phage-encoded methyltransferase, *yptb1797*) have been experimentally demonstrated to exclude other phage and plasmid DNA [53], it is conceivable that they similarly inhibit Tn*5* integration.

### Comparison with *Y. pestis* CO92 essential genes

We identified a similar number of essential in vitro growth genes in *Y. pseudotuberculosis* as those found in *Y. pestis* by Yang et al. [26], and most of these genes were orthologous. These represent conserved genes whose importance has remained vital despite substantial genomic divergence by *Y. pestis*; they include genes encoding ribosomal proteins, DNA polymerase and topoisomerase. Surprisingly, both species required genes encoding colicin import proteins (*tolA/B/R/Q*) for in vitro replication. These periplasmic toxin transporters have been described as having a role in outer membrane stability [54, 55], and for this reason may be required for optimal bacterial fitness, particularly when cultured in competition with bacilli that possess functional copies of these genes.

Intriguingly, not all essential genes from *Y. pseudotuberculosis* have orthologues present in *Y. pestis*, or they exist only in the form of pseudogenes. Conversely, *Y. pestis* also possesses unique essential genes. While comparing data from two individual labs demands caution, the fact that we matched our growth conditions suggests the broad trends we reveal warrant further examination.

Since the core processes that are required for cell viability are conserved, the unique essential genes detected from our in vitro assay tended to occur in genes involved in metabolic function. Some of these species-defining differences have already been described in *Yersinia spp.* For example, *Y. pestis* has undergone host specialisation resulting in reduced metabolic flexibility [8] with consequences including the loss of ability to metabolise rhamnose and meliobiose, and the inability to synthesise L-isoleucine or L-methionine (reviewed by Heroven and Dersch [56]). Such changes, arising from in vivo adaptation, alter the relative importance of orthologous metabolic genes, which can be detected in vitro.

In the present study, we identified *yptb0245*, a putative temperature-sensitive transcriptional repressor important in cell division [57] that is not conserved in *Y. pestis* [7]. We also identified *yptb2201*, a predicted sodium-proton antiporter with a role in salt and pH stress tolerance that is absent in *Y. pestis*. This is in agreement with the findings of Pouillot et al., who show that *yptb2201* is part of a cluster of genes that are required for optimal growth of *Y. pseudotuberculosis* at 28 °C, but have been lost during the emergence of *Y. pestis* [58]. It is possible that the loss of *yptb2201* may be compensated for by the *nhaA/B* genes, which similarly have a role in sodium-proton anti-port and are essential for the virulence of *Y. pestis* [59].

Interestingly, the two species had unique in vitro essential genes involved in LPS modification suggesting different, but equally vital cell-wall and O-antigen biosynthesis pathways. This reflects published phenotypic differences between the two species, such as the rough-type LPS produced by *Y. pestis* versus the smooth-type LPS of *Y. pseudotuberculosis* [60, 61]. Genetic shuffling of O-antigen biosynthesis genes during species divergence has similarly been reported in TraDIS comparisons between the essential genes of the closely related *Salmonella* serovars, Typhi and Typhimurium [36].

Both *Yersinia spp.* have essential genes relating to the phosphotransferase system (PTS), a translocation system responsible for the uptake of various carbohydrate substrates. However, while *Y. pseudotuberculosis* has an essential PTS gene specific to N-acetylgalactosamine uptake (*yptb3081*), a glycan particularly associated with intestinal mucosal surfaces [62], *Y. pestis* has a glucose-specific PTS component (*ypo2995*), appropriate for a pathogen adapted to replicating in the blood, where glucose is the dominant sugar available. These differences may also be explained by the fact that glucose uptake via PTS is potentially toxic for many enterobacteriacae due to the deleterious accumulation of glucose-6-phosphate [56].

Interestingly, we detected five LysR/DeoR transcriptional regulators (*yptb1518*, *yptb1927*, *yptb2301, yptb2533* and *yptb3075*) uniquely essential to *Y. pseudotuberculosis*, suggesting a non-redundant role in metabolism that is of diminished importance in *Y. pestis*. Similar regulators have been reported in *Y. pestis* as being conditionally upregulated in the flea, including *rovM* [63] and *yfbA*, which is required for biofilm formation and colonisation of the insect vector [64], but no such regulators were required for in vitro growth according to the published gene set [26].

### Genes for oxidative phosphorylation and iron acquisition are differentially essential in *Y. pseudotuberculosis* and *Y. pestis* under similar in vitro growth conditions

While haeme-synthesis genes were found to be essential to both *Y. pseudotuberculosis* and *Y. pestis* in vitro, the requirement for iron-uptake mechanisms differed. We identified four essential genes encoding iron transporters: *yptb3767* (*feoA*)*, yptb1939, yptb3227* and *yptb1343* in *Y. pseudotuberculosis* that were not essential in *Y. pestis*. These genes encode proteins for the transport of both ionic ferrous iron (*yptb3767* and *yptb1939)* and ferric iron-binding siderophores (*yptb3227* and *yptb1343*). It is interesting that these should become apparent during in vitro culture where iron is not limited, and it suggests a degree of inflexibility in which pathways are utilised between the species. Similar differences in essentiality have been noted previously: mutation of the haeme uptake gene, *hmuS* is not deleterious to *Y. pestis*, but mutation of its orthologue in *Y. enterolitica* is lethal [65]. Meanwhile, Barquist and colleagues used TraDIS to identify differences in gene essentiality between the mainly enteric *S.* Typhimurium and the mainly blood-colonising serovar, *S.* Typhi relating to iron acquisition [36]. Perhaps such differences represent specialisation to alternate microenvironments, but additional factors such as disruption of certain iron acquisition systems by mobile insertion elements may have enforced the evolution of diverse iron transporters [65].

The essentiality of *yptb3227* and *yptb1343* in strain IP32953 was unexpected, for whilst *Y. pseudotuberculosis* is known to encode several siderophores, they are not all present in every strain, and many are non-functional [66]. Yersiniabactin is carried by some strains on a mobile pathogenicity island, and other highly-virulent strains possess the yersiniabactin homologues, pseudochelin and yersiniachelin [66], but they are not required for replication in the gut. However, siderophore production during enteric infection in other species has been shown to be particularly important during the host inflammatory response (reviewed by Kortman et al. [67]). BLAST analysis of eleven additional strains of *Y. pseudotuberculosis* confirmed that *yptb3227* and *yptb1343* are chromosomally conserved, suggesting these siderophores in particular are of special importance for fitness.

Differences in iron availability and acquisition in the contrasting biological niches occupied by *Y. pestis* and *Y. pseudotuberculosis* are well documented. In vivo, *Y. pseudotuberculosis* is adapted to scavenging iron in a variety of different states through diverse, non-redundant pathways in the mammalian gut [68]. By contrast, *Y. pestis* does not require replication in the gut, and encounters mainly haeme-, lactoferrin- or transferrin-bound iron in the blood [69]. In bubonic [65] and pneumonic forms of plague, as well as in blood-plasma, ferric iron transport enabled by siderophores plays a more prominent role than ferrous iron transport [65, 70]; whilst in the flea, iron transport systems are not highly expressed [71, 72]. *Y. pseudotuberculosis* similarly upregulates ferric-binding siderophores when cultured in blood-plasma [73]. During aerobic growth in vitro, where ferric iron is freely available, similar to our experimental conditions, yersiniabactin is the favoured route of iron uptake by *Y. pestis* [74]. However, whilst yersiniabactin is required for virulence in peripheral tissue, it is dispensable in the bloodstream [75], as are the haeme transporters, Hmu and Has [76, 77]. *Y. pestis* may therefore have become a siderophore specialist, encoding many such transporters and providing a high degree of redundancy that is not the case for *Y. pseudotuberculosis* [78].

Besides the siderophores, ferrous-iron transporters including *yptb3767* (*feoA)* were essential to *Y. pseudotuberculosis* but not to *Y. pestis*. During aerobic growth of *Y. pestis*, both ferric and ferrous iron uptake systems are generally repressed by the Fe-*fur* regulon; the exception being the *feo* system, which is repressed in the presence of iron only in microaerophilic conditions [79]. *Feo* may therefore be expected to be equally important to *Y. pestis* as to *Y. pseudotuberculosis* during iron-replete aerobic growth, but its mutation is known to be compensated for by the *yfe* system. While *Y. pseudotuberculosis* also possesses the *yfe* system, both it and the *feo* system may be under different transcriptional regulation than in *Y. pestis*, altering the significance of these genes under similar aerobic and nutritional conditions.

The influence of oxygen saturation on iron acquisition regulation is particularly interesting since comparison of the TraDIS data also revealed the unique dependence of *Y. pestis* on genes involved in the tricarboxylic acid (TCA) cycle and oxidative phosphorylation. These included members of the *suc*, *sdh*, *nuo* and *cyo* operons, as well as genes encoding ATP synthase subunits and components of the electron transport chain [26, 33]. This suggests that unlike *Y. pseudotuberculosis*, *Y. pestis* favours aerobic respiration under similar in vitro growth conditions. This may again reflect its adaptation to replication in the blood stream, which is nutritionally rich and highly oxygenated compared with the gut [80]. Since there is potential cross-talk in the transcriptional and metabolic pathways that are influenced by the presence of both oxygen and iron [81–86], the differing response of the two *Yersinia* species to available oxygen may in turn affect the regulation and importance of different iron acquisition systems.

## Conclusion

By constructing a transposon library in *Y. pseudotuberculosis* and utilising TraDIS, we have been able to present the essential gene set for this species under defined growth conditions. As well as providing a resource for understanding its biology, we also demonstrate its utility in revealing new targets for the development or repurposing of existing antimicrobials, and report for the first time the sensitivity of *Y. pseudotuberculosis* to the deformylase inhibitor, actinonin. Comparison of our TraDIS data with those from a similar study performed with *Y. pestis* allowed us to identify fundamental differences in the importance of genes between *Y. pestis* and *Y. pseudotuberculosis*. Our results suggest that the two closely related species do not just favour, but depend upon, different iron uptake and metabolic pathways for survival, reflecting their adaptation to different microenvironments.

## Methods

### TraDIS library preparation

The Ez-Tn*5* Kan2 transposome complex (Epicentre, UK), was electroporated into fresh electro-competent *Y. pseudotuberculosis* IP32953 (a kind gift from Prof Richard Titball, Exeter University) at 1800 V and recovered at 28 °C in super-optimal broth with catabolite repression (SOC) media for two hours before spreading onto Yersinia selective agar base (Oxoid, UK) infused with kanamycin at 50 μg ml^− 1^. Resulting colonies, each representing unique transposon clones were removed from the agar and pooled in approximately 650 mutant batches in 40% glycerol until a total library size of about 40,000 mutants was achieved, and stored at − 80 °C. Transposon integration was validated by Southern blot and Linker PCR using standard methodology (data not shown).

In preparation for sequencing, the TraDIS library was thawed on ice and pooled together prior to genomic DNA extraction (ArchivePure, 5 Prime), or used to seed sterile Oxoid blood agar base no.2 media (BAB) (ThermoScientific) for growth at 28 °C overnight for essential gene identification experiments.

Extracted genomic DNA was fragmented by sonication and size-selected by gel electrophoresis. Gel extracted DNA was assessed for size, purity and concentration by Bioanalyser™. The fragmented gDNA was end-repaired using Klenow DNA polymerase (New England Biolabs, NEB) and T4 DNA polymerase (NEB) prior to single-primer adenylation PCR of fragments containing the transposon insert [30]. This method uses a blend of A-tailing and high-fidelity enzyme to enrich transposon-positive fragments without the need for excessive PCR cycles that may introduce amplification bias. Adapters P5 and P7 were then ligated onto the A-tailed transposon-containing fragments to serve both as the template for the sequencing primer and to allow annealing to the flow cell. Finally, samples were PCR-amplified using primers specific for the P5 and P7 adapters, with a maximum of ten cycles. Prepared DNA was sequenced using Illumina MiSeq technology.

### Sequence analysis

Raw sequencing data from three independent repeats in FASTQ format were analysed for the presence of the terminal ten base pair nucleotides of the transposon sequence with no mismatches, and then the transposon sequence was trimmed using a custom PERL script. Reads were curated for quality control and trimmed to 36 base pairs using Trimmomatic software. Bowtie software was used to map reads against the reference genome (European Molecular Biology Laboratory accession number: BX936398) with no mismatches allowed to establish the transposon insertion sites; non-unique mapping reads were discarded.

The TraDIS toolkit [87] determines gene essentiality based on the empirically observed bimodal distribution of insertion sites over genes when normalized for gene length using a method adapted from Langridge et al. [20] and Barquist et al. [36]. Briefly, a loess curve is fit to the distribution of insertion indices and used to fit a gamma distribution curve from which log-odds ratios are generated to determine the threshold for gene essentiality. Pseudogenes, and genes that were not conserved among multiple strains as identified by BLAST were excluded as false positives (data not shown). For cross-referencing against the *Y. pestis* genome, we referred to the recently published work by Yang and colleagues [26].

### Antibiotic sensitivity testing

To test the activity of actinonin against *Y. pseudotuberculosis*, we incubated approximately 1 × 10^5^ bacteria in 96-well plate format for 24 h at 28 °C in BAB broth with a titration of actinonin (in water solution) in triplicate prior to enumeration by serial dilution and assessment of colony forming units (CFU) growth.

## Additional files

Additional file 1: **Table S1.** Essential Genes List *Y. pseudotuberculosis* IP32953 in BAB Liquid Culture 28°C. The essential genes under defined in vitro conditions were calculated from raw sequencing data (see Methods) and listed; note that phage-related ‘essential’ genes are listed separately. (XLSX 32 kb)

Additional file 2: **Table S2.** Comparison of *Y. pseudotuberculosis* In vitro Essential Genes with Published *Y. pestis* Essential Genes. The essential genes that we identified in *Y. pseudotuberculosis* were cross-referenced against the published *Y. pestis* essential genes under matched conditions (Yang et al. [26]). The shared, and unique essential genes of both species are listed. (XLSX 34 kb)

## Abbreviations

ATP: Adenosine triphosphate
BLAST: Basic local alignment search tool
CDS: Coding sequence
CFU: Colony forming units
DNA: Deoxyribonucleic acid
LPS: Lipopolysaccharide
PCR: Polymerase chain reaction
RM: Restriction modification
RNA: Ribonucleic acid
SOC: Super-optimal broth with catabolite repression
TA: Toxin-antitoxin
TCA: Tricarboxylic acid
TnSeq: Transposon sequencing
TraDIS: Transposon-directed insertion site sequencing
